# A new marine gobiid species of the genus *Clariger* Jordan & Snyder (Gobiidae, Teleostei) from Taiwan

**DOI:** 10.3897/zookeys.199.2645

**Published:** 2012-06-04

**Authors:** Nian-Hong Jang-Liaw, You-Hai Gong, I-Shiung Chen

**Affiliations:** 1 Institute of Marine Biology, National Taiwan Ocean University, Keelung 20224, Taiwan, ROC; 2 CMBB, National Taiwan Ocean University, Keelung 20224, Taiwan, ROC

**Keywords:** Fish fauna, fish taxonomy, marine fish, new goby, Taiwan

## Abstract

A new species of *Clariger* Jordan & Snyder, 1901 was collected from northern Taiwan. The genus was previously known only from Japanese waters. This discovery is the first formal and southernmost record of these marine gobies from the waters of subtropical Taiwan. The new species, *Clariger taiwanensis* **sp. n.**, is distinguished from its congeners by a unique combination of features: (1) fin rays: dorsal-fin rays III, I/8; anal-fin rays modally I/8; and pectoral-fin rays modally 19 (2+16+1); (2) longitudinal dermal ridge on head with 6 barbels; and (3) specific coloration pattern: head and trunk dark brown with scattered pale spots and blotches; cheek, ventral portion of head sometimes pale with deep brown spots; pectoral-fin base with a dark brown band; and caudal fin mostly dark brown proximally and with alternating and irregular dark brown and pale bands distally. A diagnostic key to all nominal species from Japan and Taiwan is provided.

## Introduction

The generally small body-sized gobiid fishes of the family Gobiidae constitute the most diverse group of marine teleost fishes (Miller 1988; Chen and Kottelat 2005). Within the family, the *Luciogobius* generic complex includes three related genera: *Luciogobius* Gill (1859), *Astrabe* Jordan and Snyder (1901) and *Clariger* Jordan and Snyder (1901), which share the following features (Akihito et al. 2000; 2002): slender to elongate body with 30 or more vertebrae in most species, a longitudinal infraorbital papillae pattern, and first dorsal fin with fewer than 4 spinous rays or first dorsal fin absent. These gobies mainly inhabit coastal waters of Japan (Akihito et al. 2000; 2002), but their ranges also extend toward Korea, eastern China, and Taiwan (Chen and Fang 1999; Akihito et al. 2000; 2002; Wu et al. 2009). Yamada et al. (2009) investigated the molecular phylogenetics of the *Luciogobius* generic complex based on the Japanese species and revealed the very close relationship of these three gobiid genera.

One member of the *Luciogobius* complex, the coastal gobiid genus *Clariger*, has been recognized as an endemic genus of Japan that includes at least 5 nominal species: *Clariger cosmurus* Jordan and Snyder (1901), *Clariger exilis* Snyder (1911), *Clariger papillosus* Ebina (1935), *Clariger sirahamaensis* Sakamoto (1932), *Clariger chionomaculatus* Shiogaki (1988) (Shiogaki 1988, Eschmeyer and Fricke 2011) and one undescribed species, *Clariger* sp., that was recognised in Akihito et al. (2000, 2002).

*Clariger* is characterized by its cylindrical body and 3-spined first dorsal fin, which separates it from *Luciogobius*, which lacks a first dorsal fin (Akihito et al. 1984, 2000, 2002). The major differences between *Clariger* and *Astrabe* are the following features: only 1-2 free filamentous rays on the upper part of the pectoral fin in *Clariger* vs. 5-6 rays in *Astrabe*; pectoral-fin rays 18-20 vs. 24-27; and a slender body profile, rather similar to that of *Luciogobius*, vs. robust body profile in *Astrabe* (Jordan and Snyder 1901, Akihito et al. 1984, 2000, 2002; Shiogaki 1988).

Recently, collections of marine gobiid fishes have been made by the National Taiwan Ocean University (NTOU) team in the coastal region of northern Taiwan. An unusual dark goby species taken in one of these surveys appears to be an undescribed species and the first appearance of the Japanese endemic genus *Clariger* in Taiwan. The aim of this paper is to document the first record of *Clariger* from Taiwan and to describe this endemic species as new to science. A diagnostic key to all 6 nominal species of *Clariger* including the undescribed Japanese congener from Japan and Taiwan is also provided.

## Materials and methods

All type specimens of the new species were collected by hand-net. All counts and measurements were made from specimens stored in 70% ethanol after 10% formalin preservation. Morphometric methods are from Miller (1988), and meristic methods follow Akihito et al. (1984). Terminology of cephalic sensory canals and free neuromast organ (sensory papillae) is from Wongrat and Miller (1991), based on Sanzo (1911). Type specimens are deposited in the collections of National Taiwan Ocean University (NTOUP), Keelung; and National Museum of Marine Science and Technology (NMMST), Keelung.

Abbreviations: A, anal fin; C, caudal fin; D1 and D2, first and second dorsal fins, respectively; P, pectoral fin; V, pelvic fin; and VC, vertebral count. All fish lengths are standard length (SL).

## Systematics

### Clariger taiwanensis sp. n.

urn:lsid:zoobank.org:act:99B5D142-862B-4E18-959A-0A386FDEC137

http://species-id.net/wiki/Clariger_taiwanensis

Figs 1 2

#### Type material.

Holotype: 28.3 mm SL, Taiwan, Keelung City, Chau-Jin Park, 25°8.48'N, 121°48.140'E, tidal pool, 31 May 2011, Y. H. Gong (NTOUP-2011-11-062).

Paratypes: 1 specimen, 29.0 mm SL, same locality as holotype, 5 June 2006, I-S. Chen (NTOUP-2006-06-156). 5, 26.4–35.5 mm SL, Taiwan, New Taipei City, Yeliu, 25°12.08'N, 121°41.62'E, tidal pool, 7 November 2000, S. C. Wang et al. (NMMSTP 01302). 1, 30.0 mm SL, same locality as holotype, 1 November 2011, Y. H. Gong (NTOUP-2011-11-057). 1, 30.9 mm SL, Taiwan, New Taipei City, Aodi, 25°3.25'N, 121°55.81'E, tidal pool, 1 July 2011, tidal pool, Y. H. Gong et al. (NTOUP-2011-11-059).

#### Diagnosis. 

*Clariger taiwanensis* can be distinguished from other congeners by the following unique combination of features: (1) fin rays: dorsal-fin rays III, I/8; anal-fin rays modally I/8; and pectoral-fin rays modally 19 (2 free +16+1 free); (2) head with longitudinal dermal ridge including 6 barbels; and (3) specific coloration pattern when alive: head and trunk dark brown with scattered pale spots and blotches; cheek, ventral portion of head sometimes pale with deep brown spots; pectoral-fin base with a dark brown band; and caudal fin mostly dark brown proximally and with alternating and irregular dark brown and pale bands distally.

#### Description.

**Figure 1.**
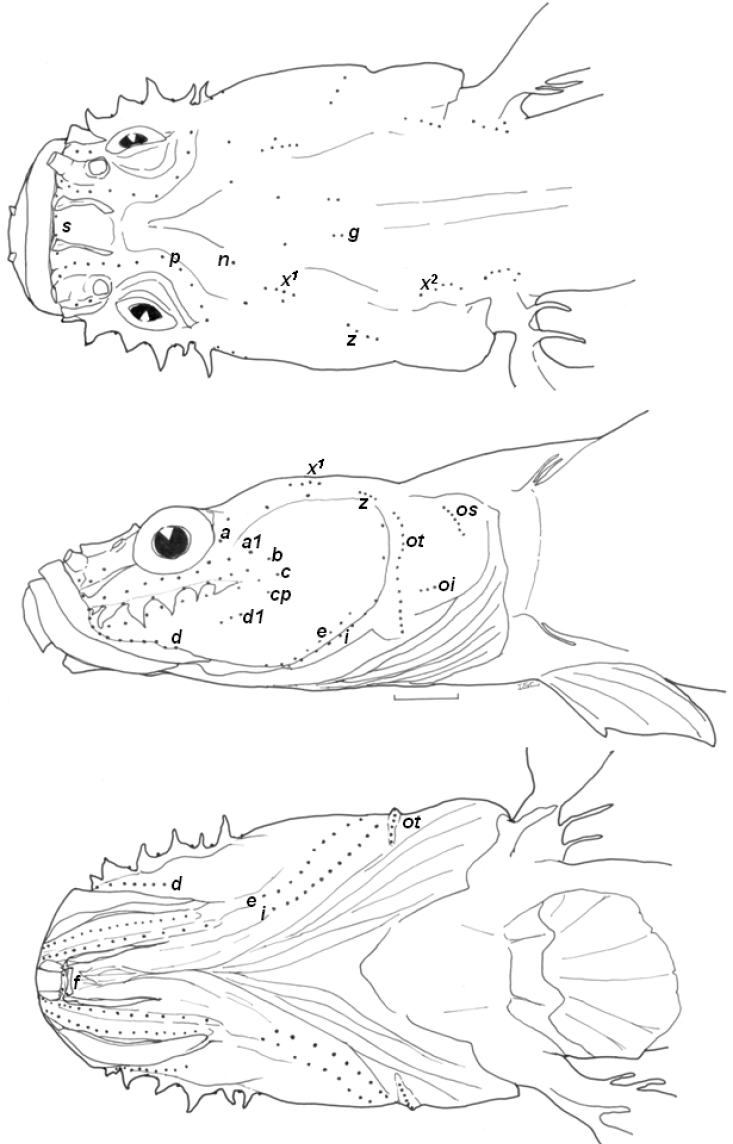
Head lateral-line system of *Clariger taiwanensis*, NTOUP-2011-11-062, holotype, 28.3 mm SL

Body rather slender, cylindrical anteriorly and laterally compressed posteriorly (all morphometric data are shown in Table 1). Head flat and depressed. Eye small. Interorbital region wide, bony interorbital width more than twice diameter of eye. Horizontal, infraorbital dermal ridge on upper part of cheek with 6 barbels (Fig. 1).

Snout flat and rather short. A pair of distinct longitudinal dermal folds beside nasal tubes on snout. Anterior nasal opening in forward-facing short tube, and posterior nasal opening round, flat. Mouth rather large, maxilla extending to vertical through rear margin of orbit. Teeth minute, jaws with 3–5 rows of conical teeth, teeth in outer rows largest in both jaws. Tongue margin bilobed anteriorly. Gill opening somewhat restricted, extending only slightly below lower margin of pectoral-fin base. Anus located anterior to vertical through origin of second dorsal fin. VC 14 + 18 = 32 (9 specimens). Trunk and head entirely naked.

*Fins*: D1 III (9 specimens); D2 I/8 (9); A I/8 (8)or I/9 (1); P 19 (2 + 16 + 1) (8) or 20 (2 + 17 + 1) (1). D1 very short in height and length. D2 and A of similar size and shape. A origin just in front of D2 origin. D2 origin on vertical between 1st and 2nd branched rays of A. P small and rounded, its length about equal to postorbital length, with 2 thin free, filamentous rays dorsally and 1 free, filamentous ray ventrally. C rounded. V with round sucking disc with complete frenum.

*Head lateral-line system*: Head canals: head lacking sensory canals and head pores (as for genus). Sensory papillae: all infraorbital sensory papillae arranged in longitudinal pattern. Row *a* long and extending forward to below nostrils. Row *b* extending from anterior region of dermal ridge to posterior region of cheek. Row *c* long, running below the dermal ridge. Single *cp* located near row *c*. Row *d* located just above upper lip, row *d1* well separated from row *d*. Rows *ot* and *oi* well separated. Row *p* surrounding orbit in interorbital region.

*Colouration in fresh and preserved specimens*: Head and trunk mostly dark brown with scattered pale spots and blotches when alive. Some individuals with larger pale spots in ventral half of trunk. Cheek, ventral portion of head, and underside of anterior portion of trunk sometimes pale with deep brown spots. First dorsal fin translucent with brown dotted spinous rays. Pectoral and second dorsal fins translucent with small deep brown spots. Pectoral-fin base with a dark brown band. Anal fin translucent with a few dark brown spots mostly on the branched rays. Caudal fin mostly dark brown proximally and with alternating and irregular dark brown and pale bands distally.

Long preserved specimens with similar overall dark pattern as described above except disappearance of body pale spots.

#### Distribution.

The new species has only been found from the coastal regions of Taipei County as well as Keelung City, Taiwan. It is highly likely that it represents an endemic marine gobiid species of Taiwan (Fig. 3).

#### Habitats.

*Clariger taiwanensis*was found in tidal pools with gravel on the rocky substratum of northern coast of Taiwan. The habitat also supports other marine gobies, including several *Bathygobius* spp. (dominant), *Eviota* spp, *Gobiopsis* spp. and *Priolepis semidoliata*, as well as the *Luciogobius* spp.

#### Etymology.

The specific name, *taiwanensis*, is in reference to the type locality from the coastal rocky shores of northern Taiwan.

#### Remarks.

*Clariger taiwanensis* shares a morphological similarity (infraorbital dermal ridge with several distinct cheek barbels) with the following four species: *Clariger cosmurus*, *Clariger exilis*, *Clariger papillosus*, and *Clariger chionomaculatus*. Cheek barbels are lacking in *Clariger sirahamaensis*. In addition to differences in cheek barbels, the new species can be separated from *Clariger sirahamaensis* by the pattern of pigmentation on the caudal-fin: fin dark brown proximally with dark brown and pale bands distally in *Clariger taiwanensis* vs. fin uniform grayish black with a pair of white spots on basal regions of both upper and lower lobes in *Clariger sirhamaensis*. *Clariger taiwanensis* can be separated from *Clariger chionomaculatus* by the number of free pectoral-fin rays (2 free rays dorsally vs. 1); and coloration (no large white marks on trunk vs. several large, round white marks on upper half of trunk). *Clariger taiwanensis* can be distinguished from the remaining three nominal species, *Clariger cosmurus*, *Clariger exilis*, and *Clariger papillosus* by the following features: (1) fin-rays counts: second dorsal-fin rays 8 vs. 10–13 in others; anal-fin rays 8 vs. 9–11; and (2) squamation: body entirely naked vs. body scaled at least on caudal peduncle.

So far as is known, *Clariger taiwanensis* is the only species of *Clariger* found outside Japan, now known from the subtropical island of Taiwan. It is very essential to survey more comprehensively the coastal waters of Taiwan as well as southern China to obtain a better understanding of the species diversity, distribution, and evolutionary history of *Clariger* and other members of the *Luciogobius* complex.

**Table 1.** Morphometry of *Clariger taiwanensis* sp. n. from Taiwan

**Type status**	**Holotype**	**All type specimens**			
**Sample No.**	**1**	**9**			
		**min**	**max**	**mean**	**standard deviation**
Total length	34.4	30.3	41.0		
Standard length	29.3	26.4	35.5		
In SL (%)					
Head length	24.4	22.8	26.7	25.3	1.2
Snout to 1st dorsal fin origin	50.0	43.6	50.0	45.8	2.1
Snout to 2nd dorsal fin origin	63.4	61.7	65.6	63.4	1.3
Snout to anus	63.5	56.6	63.5	59.8	2.3
Snout to anal fin origin	64.5	60.3	65.5	63.1	1.7
Caudal peduncle length	21.8	20.4	24.2	21.9	1.4
Caudal peduncle depth	9.9	9.9	12.5	11.1	0.8
1st dorsal fin base	4.0	4.0	7.1	5.3	1.0
2nd dorsal fin base length	16.6	15.1	19.0	16.7	1.3
Anal fin base length	16.6	15.4	19.4	16.9	1.5
Caudal fin length	19.6	14.7	19.7	17.1	1.9
Pectoral fin length	13.6	13.1	16.8	14.6	1.3
Pelvic fin length	9.7	9.7	12.5	11.2	1.0
Body depth at pelvic fin origin	11.0	10.4	11.0	10.7	0.2
Body depth at anal origin	11.5	11.4	13.5	12.6	0.8
Body width at anal origin	8.5	8.5	12.2	10.3	1.1
Pelvic finorigin to anus	35.2	30.9	38.0	33.9	2.2
Gap between bases of two dorsal fins origin	16.5	16.5	20.3	18.4	1.3
In HL (%)					
Snout length	23.8	21.3	25.4	23.5	1.3
Eye dismeter	14.1	11.3	14.3	13.3	1.2
Postorbital length	67.4	63.6	69.9	67.4	2.3
Interorbital width	16.5	14.4	19.6	16.3	1.5
Head width	54.1	54.1	66.0	58.3	4.0
Lower jaw length	35.8	31.3	38.5	35.0	2.5

**Figure 2.**
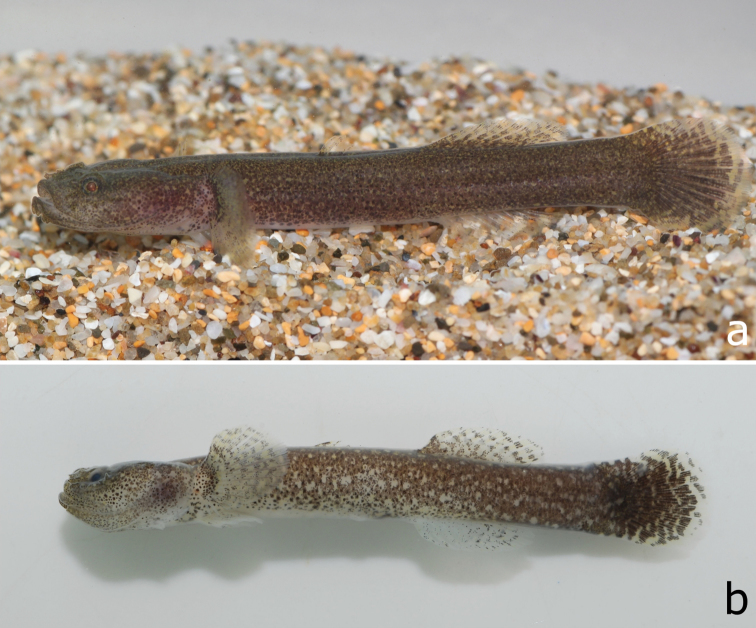
**a** A living specimen of *Clariger taiwanensis* sp. n., NTOUP-2011-11-057, paratype, 30.0 mm SL, Keelung City, Taiwan;**b** Formalin-fixed holotype, NTOUP-2011-062, 28.3 mm SL, Keelung City, Taiwan (Photograph by Kuan-Te Chen).

**Figure 3.**
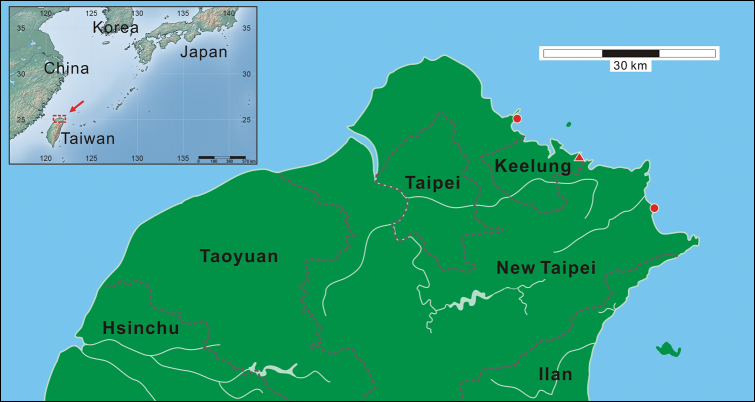
Map showing the collection localities (red symbols) for the *Clariger taiwanensis* sp. n. examined in this study in the coastal area of northern Taiwan. The red triangle shows the collection site of holotype.

#### A diagnostic key to all nominal species of *Clariger* from Japan and Taiwan (adapted from Shiogaki 1988 and Akihito et al. 2000, 2002):

**Table:** 

1a	Well-developed barbels on fleshy ridge below eye	2
1b	No barbels below eye	*Clariger sirahamaensis* Sakamoto
2a	Only one free, filamentous ray on upper region of pectoral fin; anal-fin rays modally 13	*Clariger chionomaculatus* Shiogaki
2b	Two free, filamentous rays on upper region of pectoral fin; anal-fin rays always less than 13	3
3a	Body entirely naked, second dorsal-fin rays 8	*Clariger taiwanensis* Jang-Liaw, Gong & Chen sp. n.
3b	Body with small scales at least on caudal peduncle, second dorsal-fin rays more than 9	4
4a	Gap between bases of two dorsal fins somewhat longer than body depth at anal-fin origin, head lacking distinct markings	*Clariger exilis* Snyder
4b	Gap between bases of two dorsal fins less than body depth at anal-fin origin, head with a horizontal dark stripe	5
5a	Dorsal-fin rays 10; anal-fin rays 9-10	6
5b	Dorsal-fin rays 13; anal-fin rays 11	*Clariger papillosus* Ebina
6a	Dark mark extending below the dermal barbels on ridge below eye	*Clariger cosmurus* Jordan & Snyder
6b	No dark mark below the dermal barbels on ridge below eye	*Clariger*. sp.

## Supplementary Material

XML Treatment for Clariger taiwanensis
